# Analytical Modeling of the Postcracking Response Observed in Hybrid Steel/Polypropylene Fiber-Reinforced Concrete

**DOI:** 10.3390/polym12091864

**Published:** 2020-08-19

**Authors:** Antonio Caggiano, Marco Pepe, Hernan Xargay, Enzo Martinelli

**Affiliations:** 1 Institut für Werkstoffe im Bauwesen, Technische Universität Darmstadt, 64287 Darmstadt, Germany; caggiano@wib.tu-darmstadt.de; 2 Facultad de Ingeniería, Universidad de Buenos Aires, INTECIN (UBA-CONICET), C1127AAR Buenos Aires, Argentina; hxargay@fi.uba.ar; 3 Department of Civil Engineering, University of Salerno, 84084 Fisciano (SA), Italy; mapepe@unisa.it; 4 TESIS srl, 84084 Fisciano (SA), Italy; 5 Comisión Nacional de Energía Atómica (CNEA), Departamento ICES, B1650 Buenos Aires, Argentina

**Keywords:** analytical model, hybrid fiber-reinforced concrete, steel, polypropylene, postcracking, four-point bending

## Abstract

This study deals with the analytical modeling of hybrid fiber-reinforced concretes (HyFRCs) made with a blend of different types of fibers characterized by different geometries and/or constitutive materials. The presented analytical formulation is oriented towards predicting the postcracking behavior of HyFRC and is mainly based on the well-known “cracked-hinge” model originally employed for standard fiber-reinforced concrete beams. The proposed model is validated by considering the experimental results obtained in a previous study carried out on HyFRCs mixtures made with a blend of steel and polypropylene fibers. Theoretical results are presented to demonstrate the predictive capabilities of the model to simulate the observed experimental behavior. The model performance is in very good agreement with the experimental data. Therefore, it has the capability to forecast the postcracking behavior of a generic HyFRC of given fiber contents depending on the actual proportion of the fiber blend. Finally, the proposed formulation can be applied as a computational aid to the design of HyFRC mixtures for structural purposes.

## 1. Introduction

Concrete is the most widely used construction material, mainly due to its several advantages, such as availability of constituents, cost-effectiveness, mechanical performances and low-maintenance nature [1]. Conversely, as a major drawback, plain concrete presents a brittle behavior when subjected to direct tensile and/or flexural loads [2]. Therefore, over the last decades, new cementitious composites have been studied as an alternative to the ordinary reinforced concrete with steel bars for some specific applications, such as tunnel liners, slope stabilization, ground slabs and precast concrete elements. 

The incorporation of fibers (normally ranging from 10 to 60 mm in length) within the cementitious matrix leads to a composite material generally referred to as fiber-reinforced concrete (FRC). The mechanical contribution of fibers mainly appears after crack formation, as they mitigate the intrinsic brittleness of the cement matrix by “bridging” the two sides of each crack, thus enhancing the composite toughness in the post-elastic stage [3]. Based on the material that they are made of, fibers can be classified into four groups: metallic, glass, synthetic, and natural fibers [4,5,6,7,8].

The performance improvements due to the fibers, from either the mechanical property or durability standpoint, depend on several parameters, such as volume fraction, fiber geometry, orientation and distribution [9,10,11]. Among them, the so-called aspect ratio, namely the ratio between fiber length and diameter, plays a major role as it is strictly related to the ratio between the maximum bond force that can be mobilized throughout the fiber lateral area and the maximum axial force that can be applied its transverse section [12].

The amount of fibers used in FRC is generally lower than 1.5 percent by volume but depends on the type of structural element and the target performance, with 0.5 to 1.0 percent by volume being the range most frequently used in practical applications [13].

The knowledge gained from 20 years of research activities has led to standard test methods, codes and guidelines that are currently used worldwide for the design of FRC elements [14,15,16,17,18,19,20,21,22,23].

Among the different types of fibers that can be employed in FRCs, steel ones are certainly the most commonly used [24,25]. More recently, in order to reduce the demand for raw materials and enhance sustainability in the construction industry, the use of recycled steel and natural fibers has also been investigated [26,27,28,29,30,31].

The idea of “blending” fibers with different geometric properties or materials has also been explored. These types of FRCs are often referred to as hybrid fiber-reinforced concretes (HyFRCs). Experimental results are available in the literature for HyFRC made with a combination of long and short steel fibers [32] or a blend of ordinary and recycled steel fibers [28]. Moreover, since nonmetallic fibers are also utilized [33], the mechanical response of HyFRC made with a blend of steel and polypropylene fibers can lead to synergistic effects between the two types of fiber [34,35,36,37,38].

Beyond the experimental studies mentioned above, several theoretical models and numerical procedures have been proposed in the scientific literature with the aim of predicting the bending response of FRC structural members, including their post-cracking behavior up to failure.

In principle, two alternative approaches can be followed, which are referred to as either “smeared-” or “concentrated-crack” models [39,40]. More generally, several models are also available that are based on advanced techniques, such as zero-thickness interface models [41,42], discrete particle approaches [43], X-FEM [44], E-FEM [45] and discontinuous Galerkin methods [46].

The aforementioned theoretical formulations, especially those based on discrete crack approaches employed in meso-mechanical formulations, are characterized by high predictive potential. Nevertheless, they generally require a significant computational effort. Therefore, simpler and more practice-oriented models are needed to efficiently simulate the post-cracking bridging mechanisms of fibers through concrete cracks. 

The so-called “fictitious crack” model is one of the most efficient and accurate analytical approaches available in this field [47]: it is based on assuming that bending members are controlled by the response of the most highly stressed section, where the resisting moment and the corresponding curvature are determined [48]. Hence, the assumption of a representative “hinge” length is adopted to convert curvatures into rotations and displacements (both vertical deflections and crack-open displacement, determined at either crack tip or crack mouth chord). 

Relevant evolutions of the original formulation of the “fictitious crack” model include the adoption of fundamental aspects of linear fracture mechanics [49] or a cohesive fracture model [50] also including the peculiar aspects of functionally graded FRCs [51].

However, to the best knowledge of the authors, the problem of simulating the mechanical response of HyFRC and, specifically, the mechanical modeling of FRCs with two (or more) different types of fibers is not sufficiently covered in the scientific literature.

Therefore, the present paper aims at demonstrating that the fictitious crack model can also be employed for analyzing HyFRC structural members subjected to bending actions. Specifically, a fully analytical formulation based on a multilinear axial stress–crack opening law is proposed, which is calibrated and validated by considering a series of experimental tests carried out on FRCs with different blends of polypropylene and steel fibers [36].

## 2. Model Formulation 

The formulation outlined in this section represents a further extension of the model proposed by Olesen in 2001 [47]. The latter was originally employed for analyzing the fracture behavior of FRC elements subjected to bending loads by adopting a simplified bilinear expression. 

Similar theoretical approaches, aimed at simulating the fracture behavior of either plain or FRC members, have also been proposed in the literature [49,50,51,52].

### 2.1. Constitutive Rules

The hinge model is based on the assumption that the presence of a macrocrack can locally influence the overall stress–strain fields of a frame loaded under bending. The formulation assumes that the displacement jump, created at the crack front, vanishes outside a certain width that defines the so-called “crack-hinge length”.

**Figure 1:**
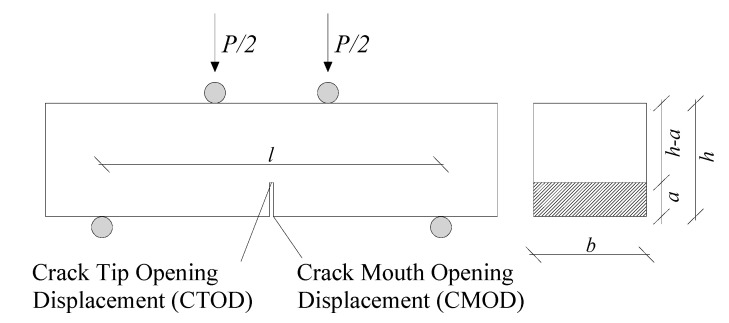
Four-point bending (4PB) test set-up.

**Figure 2:**
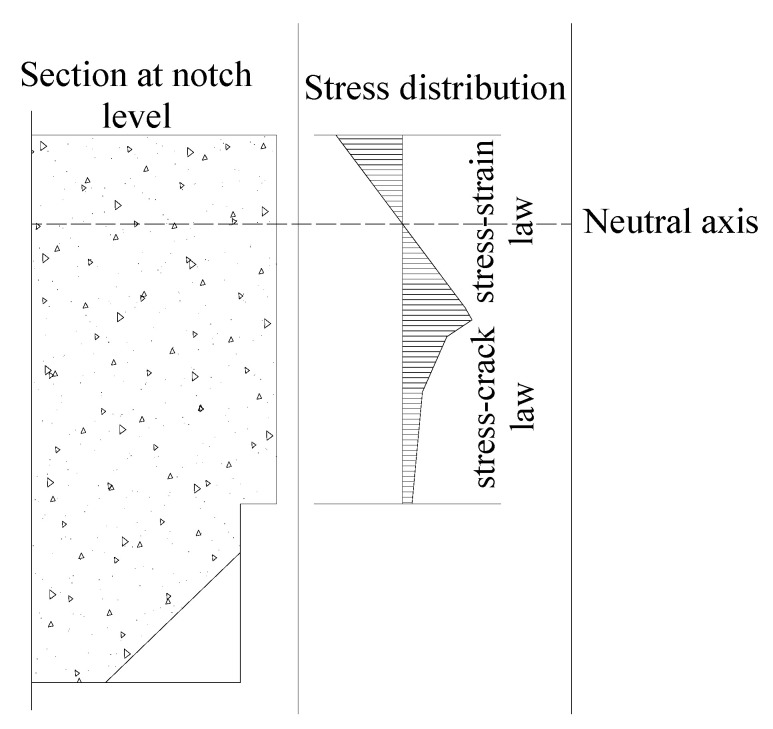
Stress distributions at the notch section.

Under this assumption, the following stress–strain and stress–crack opening relationships can be written for the hinge section (i.e., where the crack is expected to evolve) of a simulated four-point bending (4PB) test scheme (see Figure 1 and Figure 2):

(1) σ=E⋅ε

(2) $$\sigma \left(w\right)=\{\begin{array}{ccc} f_{t}-\left(f_{t}-f_{t_{1}}\right)\frac{w}{w_{1}} & \mathrm{if} & 0<w\leq w_{1}\\ f_{t_{1}}-\left(f_{t_{1}}-f_{t_{2}}\right)\frac{w-w_{1}}{w_{2}-w_{1}} & \mathrm{if} & w_{1}<w\leq w_{2}\\ f_{t_{2}}-\left(f_{t_{2}}-f_{t_{3}}\right)\frac{w-w_{2}}{w_{3}-w_{2}} & \mathrm{if} & w_{2}<w\leq w_{3}\\ f_{t_{3}}-\left(f_{t_{3}}-f_{t_{4}}\right)\frac{w-w_{3}}{w_{4}-w_{3}} & \mathrm{if} & w_{3}<w\leq w_{4}\\ 0 & \mathrm{if} & w_{4}<w \end{array}$$

where the linear elastic pre-crack state is described by the concrete modulus *E*, *ε* is the elastic strain and *σ(w)* identifies the stress–crack opening law, with *f_t_* being the concrete uniaxial tensile strength.

**Figure 3:**
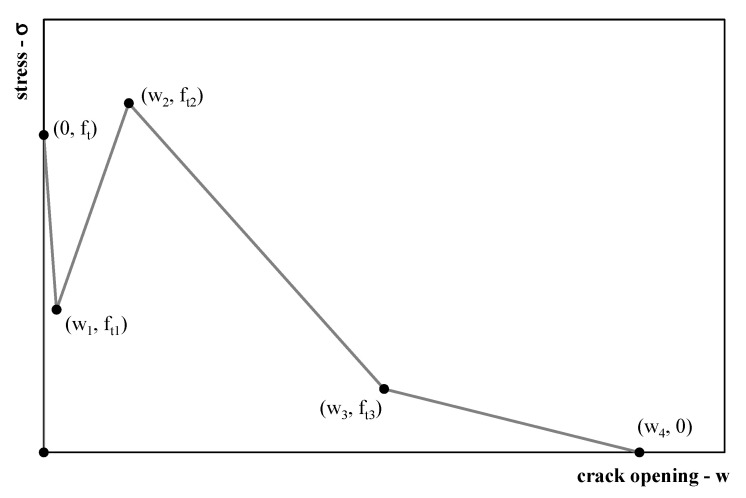
Quadrilinear stress–crack opening relationship.

A multilinear stress–crack opening relationship (also known often as softening law), following the fictitious crack model proposed by Hillerborg [48], is made via a quadrilinear shape of the stress–crack opening relationship. This shape is based on a set of four post-cracking strengths and crack opening values *w_i_*, where *i* = 1, 2, 3 and 4 (Figure 3). 

Equation (2) can also be written in compact way:

(3) $$\sigma \left(w\right)=b_{i}-a_{i}w$$

where *b_i_* and *a_i_* are the coefficients outlined in Table 1.

Under these assumptions, the total fracture energy *G_f_* (defined as the area under the softening curve as in Equation (2) can be expressed as follows:

(4) $$G_{f}=\int_{0}^{w_{4}}\sigma \left(w\right)dw=\frac{1}{2}\sum_{i=1}^{4}\left[\left(2b_{i}-a_{i}\left(w_{i-1}+w_{i}\right)\right)\left(w_{i}-w_{i-1}\right)\right]$$

### 2.2. Compatibility Equations

**Figure 4:**
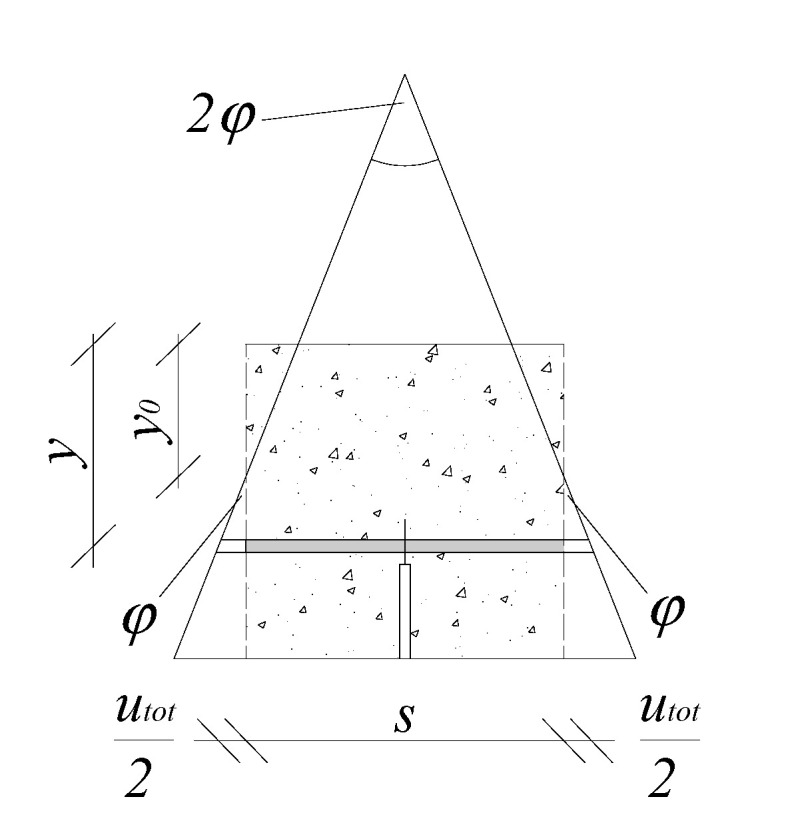
Strain at notch section and deformation of a generic strip of the hinge.

By assuming the well-known Euler–Bernoulli beam hypothesis (plane sections remain plane and normal to the axis of the beam), the mean curvature of the section k* (see Figure 4) can be evaluated as follows:

(5) $$k^{*}=\frac{2\varphi }{s}$$

where *φ* is the section rotation and *s* is the “cracked-hinge length”. The latter is assumed as a parameter of the model that needs to be duly calibrated. However, as suggested in [53], its value (for usual fiber volume contents) can be assumed as half the height of the beam.

Then, the mean value (in the crack-hinge thickness) of the longitudinal strain, ε*, can be evaluated as follows:

(6) $$\varepsilon^{*}=(y-y_{0})k^{*}$$

where *y*_0_ is the depth of the neutral axis and *y* is a generic position of the considered strip.

The total elongation of the strip *u_tot_*, in the cracked configuration and at the *y*-level, can be evaluated by means of the following adding relationship:

(7) $$u_{tot}\left(y\right)=s\varepsilon^{*}\left(y\right)=\underset{\mathrm{elastic}\;\mathrm{part}}{\underbrace{s\frac{\sigma \left(y\right)}{E}}}+\underset{\mathrm{cracking}\;\mathrm{part}}{\underbrace{w\left(y\right)}}$$

By solving Equation (7) with respect to *σ(y)*, the following equation can be obtained:

(8) $$\sigma \left(y\right)=\left(s\varepsilon^{*}\left(y\right)-w\left(y\right)\right)\frac{E}{s}=\left(2(y-y_{0})\varphi -w\left(y\right)\right)\frac{E}{s}$$

By introducing the stress–crack opening relationship of Equation (3) into Equation (8) and solving it with respect to *w(y)*, the following crack opening relationship can be obtained:

(9) $$w\left(y\right)=\frac{b_{i}f_{t}(E+a_{i}\;s\;f_{t})-E\left(\sigma +2\;a_{i}\;f_{t}(y-y_{0})\varphi \right)}{ai^{2}ft^{2}s}$$

The associated crack-bridging stress can be easily obtained by combining Equation (9) within Equation (3), obtaining

(10) $$\sigma (y)=\frac{f_{t}}{E}\left(b_{i}(E+\;a_{i}\;s\;f_{t})+2\;a_{i}\;E\;(y_{0}-y)\;\varphi -a_{i}^{2}\;f_{t}\;s\;w\right)$$

### 2.3. Equilibrium Equations

A multilayer approach has been employed, in which the hinge height *h* is subdivided in *n* number of fibers/strips (and *n* + 1 nodes) having a thickness of Δ*h* = *h*/*n*.

The position (from top to bottom) of each node is *y_i_* = *h*/2 + Δ*h_i_*_–1_. Thus, the normal force contribution from each strip *N_i_* between two nodes can be determined by the following equilibrium relationship:

(11) $$N_{i}=\left(\frac{\sigma_{i}+\sigma_{i+1}}{2}\right)\Delta h$$

where *σ_i_* is the normal stress obtainable from the constitutive rules (Equations (1) and (2)).

The eccentricity of each *N_i_*, for each strip, is based on a geometric trapezoidal calculation given as follows:

(12) $$e_{i}=\frac{1}{3}\left(\frac{\sigma_{i}+2\sigma_{i+1}}{\sigma_{i}+\sigma_{i+1}}\right)\Delta h+y_{i}$$

The moment contribution for each layer is

(13) $$M_{i}=N_{i}\cdot e_{i}$$

The sectional forces (total axial force and bending moment), with respect to *y* = 0, can be finally obtained as sums of the strip contributions:

(14) $$N_{\sec tion}=\sum_{i=1}^{n}N_{i}$$

(15) $$M_{\sec tion}=\sum_{i=1}^{n}N_{i}\cdot e_{i}$$

## 3. Summary of Experimental Results

This section summarizes the results obtained from the experimental campaign aimed at investigating the post-cracking behavior of hybrid FRC mixtures [36] prepared with either steel (S) [54] or polypropylene (P) fibers [55] or both fiber types.

It is worth mentioning that the two fiber types employed herein present different geometric and mechanical characteristics. The P fibers are a monofilament and have a nominal diameter equal to 0.032 mm and a length equal to 12 mm. The aspect ratio (*l_f_*/*d_f_*) is equal to 375. The S fibers are hooked-ended and have a diameter equal to 0.550 mm and a nominal length equal to 33 mm, leading to an aspect ratio of 60. On the other hand, the elastic modulus of the S fibers (210 GPa) is significantly higher than that of the P fibers (3.5–3.9 GPa); moreover, the tensile strength of S fibers (>1200 MPa) is more than twice that of the P fibers. 

Beyond a C25/30 reference plain concrete mixture [56], the following five HyFRC mixtures were prepared by incorporating a total volume fraction of 0.75% of blended S and P fibers in different proportions:

1. HySP-FRC-75-0, only including S fibers (S = 0.75% of the matrix);
2. HySP-FRC-55-20, with 25% of the S fibers replaced by P fibers;
3. HySP-FRC-37.5, with 50% of the S fibers replaced by P fibers;
4. HySP-FRC-20-55, with 75% of the S fibers replaced by P fibers;
5. HySP-FRC-0-75, only including P fibers (P = 0.75 % of the matrix).

Four-point bending tests were performed in accordance with UNI 11039-1 [19] and UNI 11039-2 [20] on notched prismatic specimens (see Figure 1), with the aim of characterizing the post-cracking flexural behavior of HySP-FRC samples. A total number of 18 prismatic (150 × 150 × 600 mm^3^) specimens (3 for each mixture) were prepared and tested after 28 days of curing. Three displacement transducers were placed at the bottom of the notch in order to measure their surfaces relative displacement (the so-called crack mouth opening displacement (CMOD)). The displacement rate was set to 0.005 mm/min. For each test performed, the CMOD was calculated as the average of the three measurements (CMOD_m_). 

**Figure 5:**
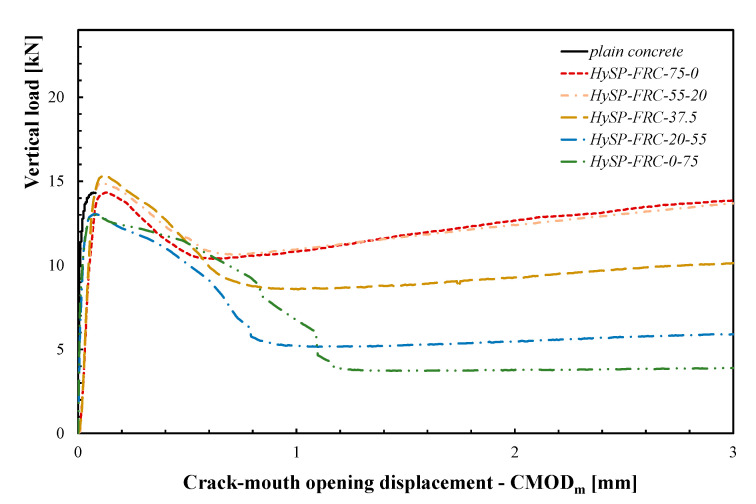
Mean vertical load–mean crack mouth opening displacement (CMOD_m_) curves for HySP-FRCs.

Figure 5 reports the mean vertical load versus the corresponding CMOD_m_ curves obtained during the tests.

The post-cracking flexural response of FRC specimens reinforced with only S fibers is characterized by a significant toughness, which is due to the bridging action of fibers and cannot be obtained in plain concrete. The effect of the gradual replacement of a given amount of S fibers with an equal volume fraction of P ones is clearly seen in the curves represented in Figure 5. It can be noticed that the post-cracking behavior of HySP-FRC is characterized by a more pronounced softening range (lower toughness) in the mixtures containing a higher volume fraction of P fibers in substitution of the S ones. This is a consequence of the lower efficiency of the P fibers with respect to the S ones, which are specifically designed to exhibit a good interaction with the cement matrix. This behavior can be mainly attributed to the existing difference in terms of geometrical and mechanical performance between the P and S fibers: the latter are characterized by a greater length and diameter and a higher elastic modulus and tensile strength in comparison with the polypropylene reinforcements. The presence of the P fibers improves the mechanical behavior immediately after the peak, in the presence of small cracks. The experimental results demonstrate that when increasing the amount of P fibers, the descending phase (the so-called softening branch) of the F–CMOD_m_ curve is longer and less steep. On the other hand, the residual strengths rapidly decreased, as fibers quickly debonded from the cement matrix. 

A more comprehensive analysis can be performed by analyzing the representative parameters defined by the UNI-11039 [19,20]:

- First crack strength (*f_lf_*):
  (16) $$f_{lf}=\frac{P_{lf}l}{h{\left(b-a_{0}\right)}^{2}}$$
  where *P_lf_* represents the first crack load (in N), *b*; *h* and *l* are the width (in mm), height (in mm) and spam length (in mm) of the tested beam, respectively; and *a*_0_ (in mm) represents the notch depth.
- Work capacity indices: *U*_1_ and *U*_2_ (energy absorption values) represent the areas under the vertical load (*P*)–CTOD curve in a representative range for the serviceability limit state (i.e., considering a CTOD ranging between CTOD_0_ and CTOD_0+0.6_) and for the ultimate state (i.e., considering a CTOD ranging between CTOD_0+0.6_ and CTOD_0.6+3_), respectively.
- Equivalent post-cracking strengths: The first one (*f_eq_*_(0–0.6)_) is supposed to be significant for the serviceability limit state (evaluated as a function of the *U*_1_ parameter), whereas the second one (*f_eq_*_(0.6–3)_) is rather relevant for the ultimate state (evaluated as a function of the *U*_2_ parameter).
- Ductility indices: *D*_0_ and *D*_1_ can be determined with the following equations:
  (17) $$D_{0}=\frac{f_{eq(0-0.6)}}{f_{lf}}$$
  (18) $$D_{1}=\frac{f_{eq(0.6-3)}}{f_{eq(0-0.6)}}$$

**Figure 6:**
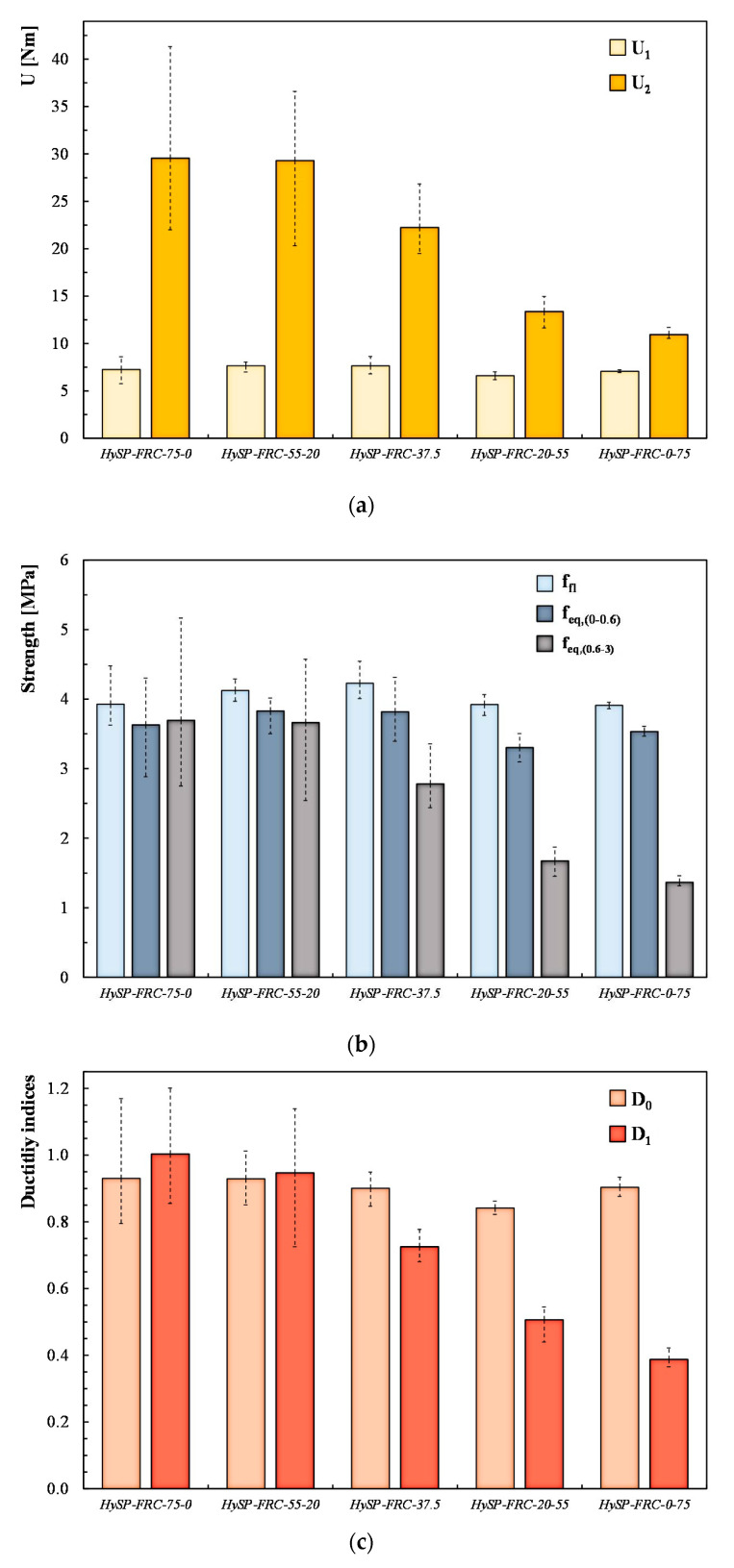
Representative parameters for tested fiber-reinforced concrete (FRC) mixtures: (**a**) work capacity indices, (**b**) first crack and equivalent strengths, (**c**) ductility indices.

Figure 6 highlights the influence of the replacement of S fibers with P ones by reporting the variation of the representative parameters described above.

The results indicate that the presence of P fibers leads to a slight increase in the first crack strength (Figure 6b). In fact, the HySP-FRC-75-0 mixture presents a first crack strength equal to 3.93 MPa, while higher values were registered for the other mixtures. This evidence can be attributed to the higher number of fibers present in the cross-section when P fibers are introduced. In fact, the S fibers are characterized by an aspect ratio significantly lower than that of the P ones (around 6 times lower).

On the other hand, the results in terms of ductility indices (*D*_0_ and *D*_1_ in Figure 6c) highlight that the presence of P fibers tends to decrease the FRC toughness. The *D*_0_ seems to be almost unaffected: it moves from 0.93 (for HySP-FRC-75-0) to 0.90 (for HySP-FRC-0-75). In terms of ductility index *D*_1_, the presence of P fibers moves the post-cracking behavior from a plastic (*D*_1_ equal to 1.0 for HySP-FRC-75-0) to a softening behavior.

The description of materials, methods and results obtained from the aforementioned tests has been kept as concise as possible in this paper. Further relevant details are reported in a previous study already available in the literature [36].

## 4. Model Validation

An inverse analysis procedure was conducted to calibrate the values of the parameters defining the quadrilinear stress–crack opening rule outlined in the previous subsection. Although previous works [21,32,47] assumed that *s* is one-half of the hinge height, it actually depends on the type of structural element and on the fiber types and volume fraction of the considered specimen. Therefore, the ratio *E*/*s* appearing in Equation (7) is assumed hereafter as a parameter to be fitted. 

The calibration procedure was performed starting from the data of each HyFRC composite. Particularly, the best fit was calibrated for the load–CMOD curves across the interval 0 < CMOD < 3 mm.

Table 2 reports, for each mixture, the adopted values of the quadrilinear stress–crack opening rule obtained from the inverse analysis.

**Figure 7:**
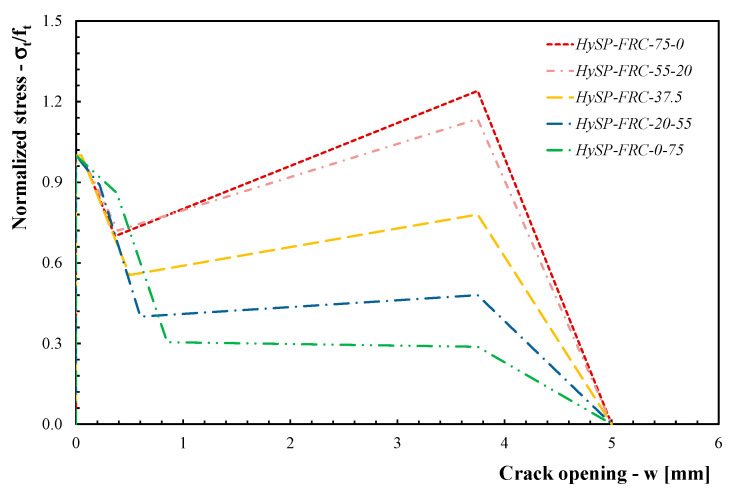
Representative parameters for tested FRC mixtures.

Figure 7 reports the stress–crack relations for the various FRC mixtures. 

**Figure 8:**
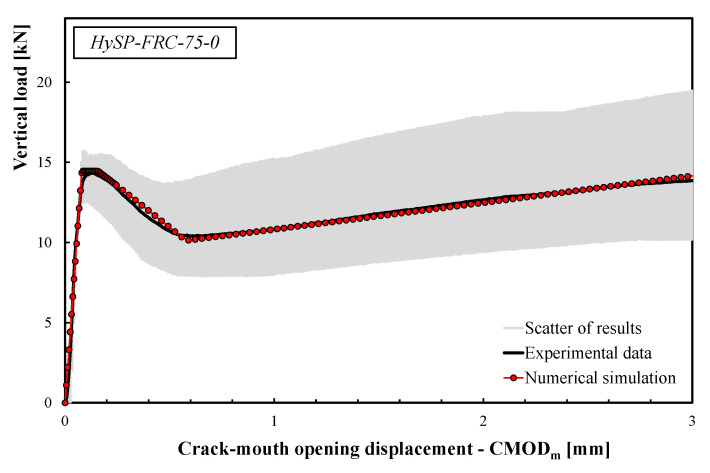
Experimental and numerical load–CMOD curves for the HySP-FRC-75-0 mixture.

**Figure 9:**
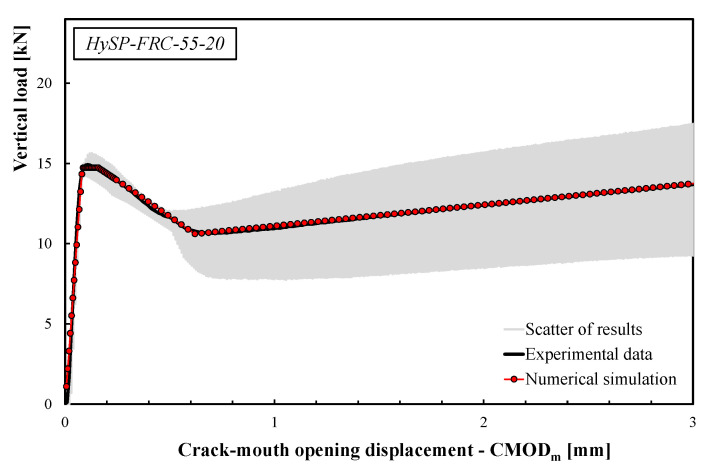
Experimental and numerical load–CMOD curves for the HySP-FRC-55-20 mixture.

**Figure 10:**
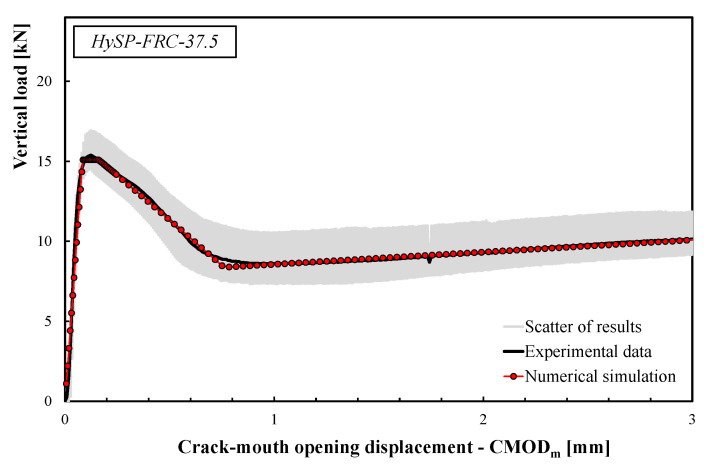
Experimental and numerical load–CMOD curves for the HySP-FRC-37.5 mixture.

**Figure 11:**
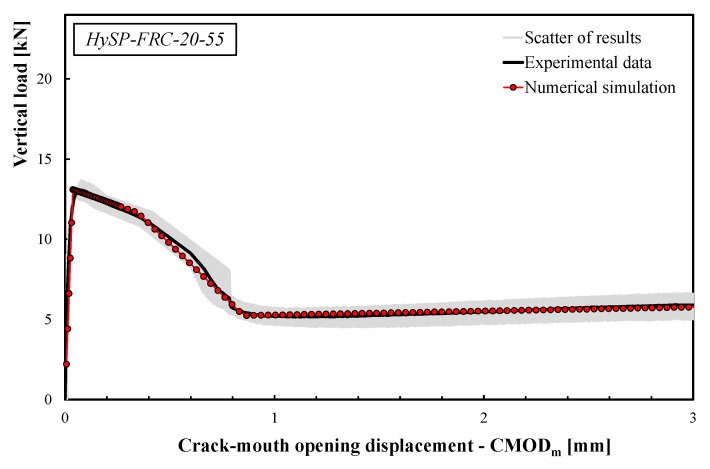
Experimental and numerical load–CMOD curves for the HySP-FRC-20-55 mixture.

**Figure 12:**
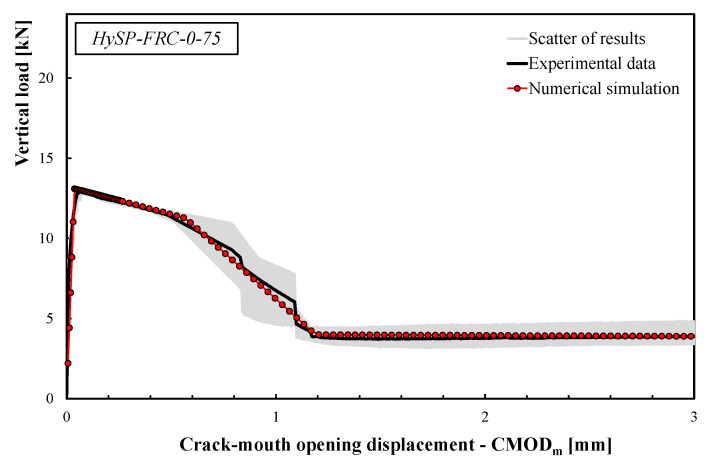
Experimental and numerical load–CMOD curves for the HySP-FRC-0-75 mixture.

The comparisons between the numerical simulations and the experimental data are presented in Figure 8, Figure 9, Figure 10, Figure 11 and Figure 12. For each mixture, the black line represents the average response of the three tests, the grey area highlights the scatter between the experimental results and the red line describes the theoretical simulation. Good accuracy of the simulated results for all specimens was obtained, demonstrating that the adopted stress–crack rules employed in the extended Olesen’s model allow obtaining load–CMOD numerical responses in very good agreement with the experimental data. 

The scatter reported by the gray area (see Figure 8, Figure 9, Figure 10, Figure 11 and Figure 12) is generally due to various phenomena/parameters, such as the irregular space distribution of fibers and/or the variable number and orientation of fibers crossing the cracking surface, which contribute to the inherent uncertainty of the experimental data, especially when composite materials are used.

The results highlight that the post-cracking responses of FRC mixtures made with only S fibers present a significant toughness as a result of the bridging action exploited by this type of reinforcement. In addition, the effect of replacing part of the S fibers with an equivalent volume of P fibers can be easily seen by analyzing the curves reported in Figure 9, Figure 10, Figure 11 and Figure 12.

As a general trend, the post-cracking behavior of HyFRC can be divided into three stages. In the first stage, the slope of the descending (softening) branch is more pronounced for the mixtures with higher content of S fibers, denoting a more delayed activation of such fibers in comparison to the P fibers: this is mainly due to the lower number of S fibers corresponding to the same volume of P ones.

In the second phase, S fibers begin to make a significant contribution, while the P ones tend to lose their action mainly due to debonding, tensile failure mechanisms or both. Therefore, on the one hand, a re-hardening response can be observed for the specimens characterized by a high amount of S fibers, while a crack-softening behavior was observed for specimens with a predominant percentage of P fibers. In the last phase, the activated P fibers have already exceeded their maximum bond strength, and they react with a constant friction force; meanwhile, a further bridging effect is still offered by S fibers, which actually results in a hardening response as the crack opening increases.

The results indicate that the overall shape of the stress–crack opening displacement curves of HyFRC significantly depends on the considered fiber types. Specimens with only P fibers were characterized by an excellent post-cracking strength for crack widths that are relevant for the serviceability limit states (i.e., *w* = 0.5 or 0.6 mm according to RILEM [22,23] or UNI 11039-1&2 [19,20] provisions, respectively). Furthermore, a significant re-hardening response during the post-cracking phase was observed in specimens with higher fractions of S fibers. On the other hand, a lower residual load carrying capacity can be appreciated for values of crack widths compatible with the ultimate limit states (*w* = 2.5–3.0 mm) when a greater volume fraction of P fibers in substitution of S fibers is considered (e.g., the *σ–w* curves of the HySP-FRC-37.5, HySP-FRC-20-55 and HySP-FRC-0-75 mixtures).

## 5. Conclusions

The present paper presents a contribution to the prediction of the post-cracking behavior of an emerging class of material generally referred to as HyFRC. Specifically, the case of FRC with a blend of steel and polypropylene fibers has been considered. The following considerations can be pointed out:

- The proposed model is based on sufficiently general, yet analytically expressed, normal stress–crack opening relationships, which can potentially simulate the various possible responses observed in experimental tests on FRC specimens.
- The model parameters were identified for each mixture, and a very good agreement was obtained between the experimental results and the simulation output for all the HyFRC specimens considered in this study.
- Moreover, the parameters obtained for the normal stress–crack opening identified for the various HyFRC specimens exhibited a regular variability with respect to the fiber content and proportions of each specimen.

Therefore, the presented results demonstrate that the model is potentially capable not only to simulate the observed experimental results, but also to predict the post-cracking behavior of a generic HyFRC depending on the actual proportion of the fiber blend. Demonstrating the possible capability of the model to infer the behavior of HyFRC based on the calibration on specimens with different fiber contents is one of the future areas for development of this research. The proposed formulation represents a tool for the design of HyFRC structural members and can also be useful for future industrial applications.

## Figures and Tables

**Table 1:** The *b_i_* and *a_i_* Coefficients of the Quadrilinear Stress–Crack Rule.

*b_i_*	*a_i_*	*w*
1	$\frac{f_{t}-f_{t_{1}}}{f_{t}\;w_{1}}$	$0<w\leq w_{1}$
$\frac{f_{t_{2}}w_{1}-f_{t_{1}}w_{2}}{f_{t}\;\left(w_{1}-w_{2}\right)}$	$\frac{b_{i}f_{t}-f_{t_{1}}}{f_{t}\;w_{1}}$	$w_{1}<w\leq w_{2}$
$\frac{f_{t_{3}}w_{2}-f_{t_{2}}w_{3}}{f_{t}\;\left(w_{2}-w_{3}\right)}$	$\frac{b_{i}f_{t}-f_{t_{2}}}{f_{t}\;w_{2}}$	$w_{2}<w\leq w_{3}$
$\frac{-f_{t_{3}}w_{4}}{f_{t}\;\left(w_{3}-w_{4}\right)}$	$\frac{b_{i}f_{t}-f_{t_{3}}}{f_{t}\;w_{3}}$	$w_{3}<w\leq w_{4}$
0	0	$w_{4}<w$

**Table 2:** Parameters of the Stress–Crack Opening Relations Obtained Through the Inverse Analysis.

Mixtures	*f_t_*	*α*_1_	*w*_1_	*α*_2_	*w*_2_	*α*_3_	*w*_3_	*w*_4_	*E/s*
	[MPa]	-	[mm]	-	[mm]	-	[mm]	[mm]	[MPa/mm]
HySP-FRC-75-0	2.650	1.000	0.050	0.700	0.363	1.240	3.750	5.000	52.213
HySP-FRC-55-20	2.700	1.000	0.050	0.720	0.390	1.135	3.750	5.000	52.213
HySP-FRC-37.5	2.765	1.000	0.050	0.555	0.500	0.780	3.750	5.000	52.213
HySP-FRC-20-55	2.400	0.890	0.220	0.400	0.600	0.480	3.750	5.000	104.425
HySP-FRC-0-75	2.400	0.860	0.380	0.305	0.850	0.288	3.750	5.000	104.425
